# How do mental health clinical placements influence pathways to practice for mental health nursing students? A systematic review

**DOI:** 10.1016/j.ijnsa.2026.100708

**Published:** 2026-10-15

**Authors:** Kristina Griffin, Samantha Jakimowicz, Alison Robinson, Andrea McKittrick

**Affiliations:** a School of Nursing, Paramedicine and Healthcare Sciences, Charles Sturt University Port Macquarie, NSW, Australia; b School of Nursing, Paramedicine and Healthcare Sciences, Charles Sturt University, Bathurst, NSW, Australia; c School of Nursing, Paramedicine and Healthcare Sciences, Charles Sturt University, Wagga, NSW, Australia; d Department of Occupational Therapy, Royal Brisbane & Women's Hospital, Herston, QLD, Australia

**Keywords:** Learning, Experiences, Autonomy, Impact, Influence, Career preference, Mentorship

## Abstract

Workforce shortages present challenges for mental health nurses. Rural and remote regions of Australia face more challenges due to workforce shortages and geographical isolation. This systematic review examines the impact and influence of clinical placements on nursing students and their perceived post graduate career preferences which is an important consideration in addressing workforce challenges. A comprehensive search of MEDLINE and CINAHL was conducted, from January 2000 to April 2026. Thirty-five studies were included in the systematic review. Forty- eight percent of studies used surveys to collect data utilizing a combination of standardized and study-specific surveys. This review also identified three key themes across all studies. ***Empowerment through supportive clinical engagement*** included the development of autonomy and confidence in nursing, opportunities for hands on clinical skills, and the benefits of supportive mentoring during placement. ***Barriers to professional growth in clinical practice*** were marked by inadequate support and training, limited learning opportunities, feeling unwelcomed and unchallenging work activities while on clinical placement. ***Practice area types*** specifically in mental health, critical care and aged care settings were associated with negative experiences, while placements in general practice, intensive care units, and structured university training modules for specific clinical placements contributed positively to students' experiences. The findings point to areas for consideration during nursing students training which may address workforce issues over time. The results support integrating specific practice area information into curriculums, offering preceptorship opportunities during placement, and exploring non-traditional placement models which may help address the perceived challenges identified by nursing student during their training. Further research is required to explore innovative placement models and strategies to optimise learning across diverse practice areas.

**What is already known**

• Workforce shortages present challenges for mental health nurses.
• Rural and remote regions of Australia face more challenges due to workforce shortages and geographical isolation.
• This systematic review examines the impact and influence of clinical placements on nursing students and their perceived post graduate career preferences which is an important consideration in addressing workforce challenges.

**What this paper adds**

• Three themes emerge from the literature on what influences post graduate employment choices for nurses : empowerment through supportive clinical engagement, barriers to professional growth in clinical practice and practice area types.
• Placement experiences were found to influence postgraduate career preferences.
• Improving placement experiences for nursing students has the potential to influence career preferences post-graduation addressing workforce shortages.

## 1 Introduction

Nursing is a global profession who provide person centred, evidence-based care to individuals of all ages with various illnesses, diagnoses and injuries (AHPRA., 2024). Multiple specialist areas of nursing practice exist across the spectrum of care (AHPRA., 2024). However limited information is published on variables which contribute to choice of post-graduate practice preferences for nurses (Jirwe and Rudman, 2012; Wu et al., 2015). Location, life experience, past experiences, current age and life goals are all factors which may contribute and change over the career of a nurse (Han et al., 2018). The importance of nursing staff in healthcare cannot be underestimated (Gatchel, 2018), however the role and scope of nursing has become more varied and complex as opportunities for practice increases (Gatchel, 2018).

Nursing is recognised as a multifaceted profession which requires the integration of clinical expertise, critical thinking, communication, and compassionate care (Gatchel, 2018). However for many years workforce shortages have existed in the field of nursing on a national and international scale (Tamata and Mohammadnezhad, 2023). Post the Global Covid 19 pandemic the strain on workforce has remained (Peters, 2023) and is more prominent in regional and rural areas (Tran et al., 2024). The lack of nursing workforce in these areas can compromise healthcare quality, choice and standards for individuals requiring medical care (Tran et al., 2024). The practice area of mental health nursing has become more prominent as increased awareness and diagnoses of mental health illnesses occur (Hurley, 2009). However, the demand for care and services outstrips the availability of mental health nurses on a national and international level (Furber et al., 2015). Specifically in Australia, there is a rise in prevalence of mental health conditions and an increased recognition of their impact on individuals and communities (Yang, 2024). One in five Australians experienced mental health issues each year therefore the need for nurses working in this area is important (Yang, 2024).

Given that all Australian nursing students undertake mental health content and clinical placements as part of their undergraduate training, these early experiences may play a critical role in shaping interest in mental health nursing as a career pathway. Understanding how clinical placements influence students’ perceptions of mental health practice is essential for strengthening the future workforce and addressing national shortages.

## 2 Background

To address workforce shortages and gaps in nursing practice, it is important to understand the variables which contribute to these issues (Peters, 2023). Burn out, lack of support and lack of resources are some contributing factors (Han et al., 2018; Peters, 2023). A significant proportion of the current nursing workforce is nearing retirement age, and there is insufficient replacement of experienced nurses with new graduates across multiple nursing specialities (Adams et al., 2021; Peters, 2023). This generational gap further exacerbates workforce shortages, as fewer younger nurses are entering the workforce including the specialty area of mental health nursing (Jirwe and Rudman, 2012; Tran et al., 2024; Wu et al., 2015). Additionally, the financial and time constraints faced by nurses to pursue further education can discourage individuals from continuing to practice nursing (Cottle‐Quinn et al., 2022). Lack of advanced training opportunities may also contribute to the insufficient pipeline of skilled professionals (Adams et al., 2021; Cottle‐Quinn et al., 2022).

Rural and remote areas of Australia face more acute shortages of nurses due to factors such as geographical isolation, limited access to professional development, and lower salaries compared to metropolitan areas (Beks et al., 2018; Liu et al., 2023). Rural and remote areas of Australia can be hundreds of kilometres from a metropolitan centre with limited transport options. This increases gaps in services in these regions and places additional strain on metropolitan health services as patients are transferred for cares (Bourke et al., 2021). This places strain on the individuals who require healthcare and also for the local services when they return to their communities (Bourke et al., 2021). Addressing these issues requires a multifaceted approach, including improved working conditions, increased investment in education and training, and strategies to increase workforce retention in nursing in Australia (Fortabong et al., 2025).

In Australia, several universities in regional and rural locations offer nursing degrees, and some of these programs are specifically aimed at training student nurses to work in mental health (Griffin et al., 2026). Mental health nursing is not offered as a standalone undergraduate specialty. All Bachelor of Nursing students complete mental health theory and clinical placements (inclusive of a mental health placement) as part of the core curriculum, making undergraduate nursing students the relevant population for examining early exposure to mental health practice. These universities often collaborate with local healthcare providers to provide students with clinical placements (both general and mental health) in rural and remote settings, helping to address the healthcare workforce shortages in these regions (Griffin et al., 2026). Individual experiences shape people’s desires to continue with roles which are meaningful to them. Early experiences and exposure to the nursing profession can influence post graduate career preferences (Griffin et al., 2026), therefore there is need to explore the current evidence base to determine how these experiences shape post graduate career preferences in mental health nursing. The aim of this study is to examine how mental health clinical placements influence undergraduate nursing students’ perceptions of mental health nursing and their subsequent post-graduate perceived career preferences.

## 3 Methods

A systematic review was chosen to provide synthesis and critical appraisal of evidence to address the aim outlined above. The PRIMSA guideline was followed (Page et al., 2021).This review was registered on the international prospective register of systematic reviews PROSPERO 2024 (CRD42024529276).

### 3.1 Search strategy

A search was undertaken of the following electronic data bases MEDLINE and CINAHL in June 2024 and April 2026 to search for relevant studies published from Jan 2000 to April 2026. These two data bases were chosen as they are considered two of the most comprehensive and authoritative sources for healthcare and nursing research. The time frame was chosen as the Covid 19 Pandemic impacted mental health everywhere, but rural and remote communities were hit especially hard for several reasons: social isolation, economic stress, limited access to healthcare and fear/ uncertainty. Since 2020, there has been a significant global increase in awareness and discourse surrounding mental health, particularly following the pandemic, which brought these issues to the forefront. The search terms used for this review are summarised in Table 1. A wide search was undertaken to ensure all research data pertaining to clinical placement experiences were captured.

**Table 1:** Search terms.

**Boolean Phrases used during the search**
(MH "Student Placement") OR (“clinical placement*" or "clinical experience*" or "workplace learning" or "recovery camps" OR (student* N3 placement*) OR (Non-traditional N4 placement*) OR “preregistration education” OR “clinical learning” OR “work-based learning” OR “workbased leaning” OR “clinical education” OR “practice placement*” OR “work integrated learning” OR “prelicensure education” OR “clinical fieldwork” OR “work practicum”) AND ((MH "Students, Allied Health") OR (MH "Students, Nursing+") OR (MH "Students, Medical+") OR (MH "Students, Occupational Therapy") OR (MH "Students, Physical Therapy") OR (MH "Students, Social Work") OR (MH “Education, Clinical”)) OR (student* N3 (health* OR nurs* OR physio* OR “occupational therap*” or “social work” OR medicine OR psychology* OR doctor* or medical or "physical therap*" or “exercise physiologist*” or “allied health”)) AND ((MH "Careers in Nursing") OR (MH "Careers in Allied Health") OR (MH "Career Planning and Development") OR (MH “Personnel Recruitment”)) OR ("pathways to practice" OR (career N3 influenc*) OR recruitment OR (career* N3 pursue) OR “clinical confidence” OR “future workplace” OR (career* N3 choice*)) AND ((MH "Student Experiences") OR (MH “Student Attitudes”) OR (MH “Student Satisfaction”)) OR ((student* N3 experience*) OR "placement experience*" OR (positive N3 experience*) OR (positive N3 influence*) or (placement* N3 impact*))

### 3.2 Inclusion criteria

Studies available in the English language and published in a peer review journal. Participants in included studies had to be nursing students who had participated in clinical (general and mental health) placements. Studies had to report quantitative or qualitative outcomes at baseline (preplacement) or follow up (post placement).

### 3.3 Exclusion criteria

Studies were excluded if they were discussion papers, conference proceedings, editorials, literature reviews, grey literature, or letters to the editor. This was to ensure that the included evidence had undergone a rigorous peer review process. Studies focusing on other undergraduate students were excluded, if no nursing cohort was reported, due to different support structures available for these programs within the Australian context.

### 3.4 Selection process

The initial search yielded 558 studies, of which 35 duplicates were removed. Two reviewers independently used Covidence ™ software for screening and full text review of potential studies. Titles and abstracts of 523 studies were screened against the eligibility criteria. Full text review was undertaken for 138 studies; discrepancies were resolved by discussion with all researchers. No additional studies were identified through comprehensive searches and review of reference lists from the included studies. The final numbers of studies included in this review was 35. (See Fig. 1 for PRISMA diagram).

**Fig. 1:**
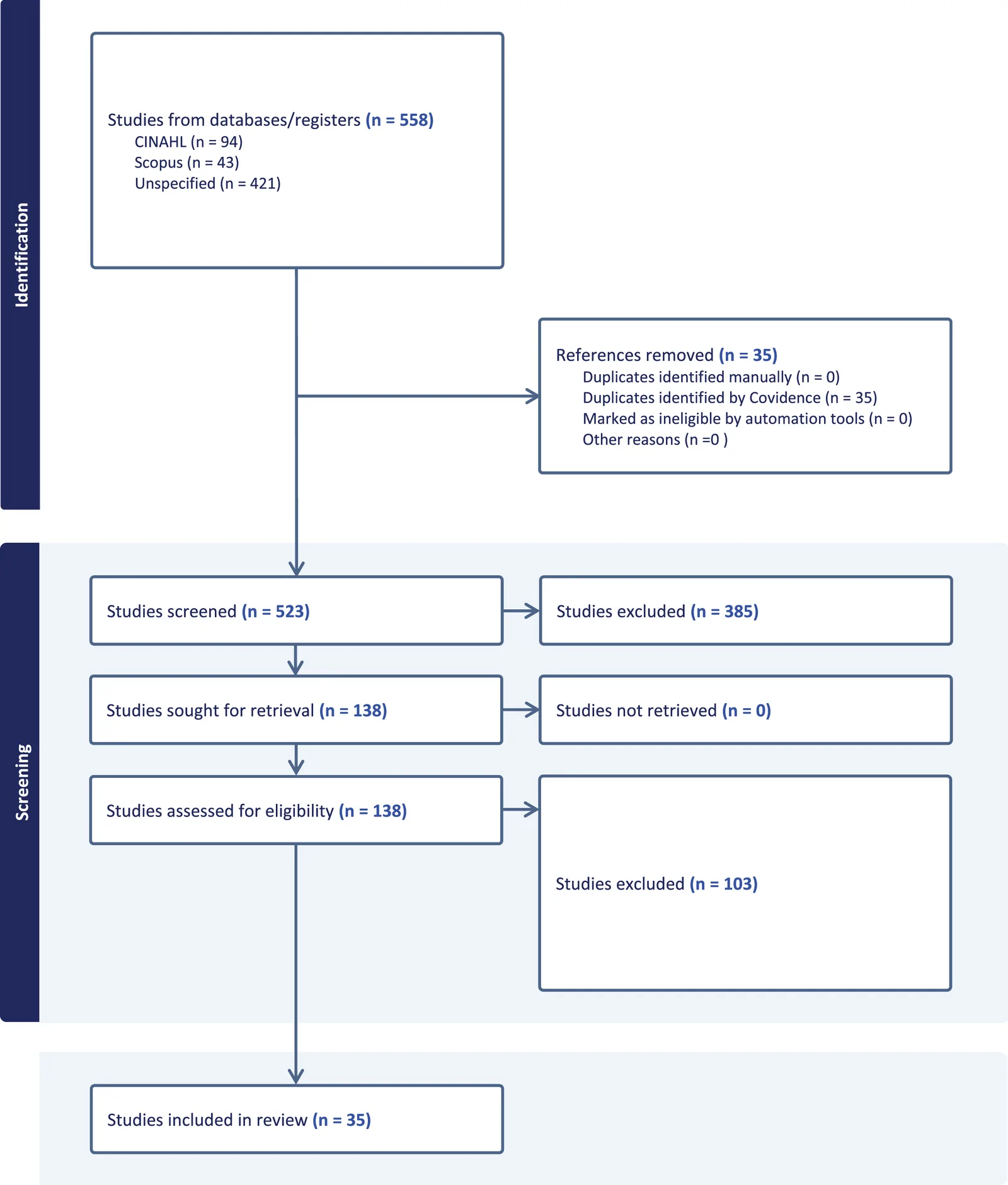
PRIMSA.

### 3.5 Data extraction

Data extraction was undertaken by two reviewers and crossed checked by a third. The following data was extracted: year, purpose of study, participants, setting, methodology and outcomes.

### 3.6 Quality assessment

1. Risk of bias and 2. evidence of validity and reliability were assessed using critical appraisal tools from the Joanna Briggs Institute, PRISMA extension for scoping reviews (PRISMA-ScR) and McGill University (Mixed Methods Appraisal Tool (MMAT) version 2018 (Hong et al., 2018). Different critical appraisal tools were required to different types of study designs being included in this systematic review. Each study was critically appraised by two reviewers independently using established critical appraisal tools. The reviewers met on eight occasions to discuss and finalise the results. Consensus reached was subsequently mapped to the relevant critical appraisal tools corresponding to the specific study design. Table 3, Table 4, Table 5, Table 6, Table 7, Table 8 (Appendix 1) summarises the quality mapping of the included studies.

### 3.7 Data analysis

A meta-analysis was not possible due to the heterogeneity of study designs included in the results (Campbell et al., 2020). The qualitative data was synthesised using a thematic analysis approach (Braun et al., 2024). Initially, two researchers engaged in familiarization with the data through repeated reading to gain an in-depth understanding of its content. This was followed by the systematic generation of initial codes, identifying and organizing significant features of the data (Braun et al., 2024). The next phase involved sorting these codes into potential themes, which were then reviewed to ensure they accurately reflected the dataset. Subsequently, each theme was clearly defined and named to capture its central meaning (Braun et al., 2024). The quantitative data was analysed using descriptive statistics.

## 4 Results

Thirty-five studies were included in this systematic review. The studies were published in Australia n = 25, Norway n = 1, Singapore n = 1, the United Arab Emirates n = 1, the United States of America n = 1, and the United Kingdom n = 6. Study designs reported were qualitative reviews n = 11, cross sectional n = 8, mixed methods n = 8, case control n = 1, quantitative n = 2, quasi experimental design n = 1, pre and post-test questionnaire n = 1, correlational design n = 1, longitudinal descriptive design n = 1 and cohort study n = 1.

Table 2 outlines the outcomes (impact and influence) for each paper included in this review. Ninety four percent of the studies focused on the experiences of undergraduate nursing students and 94% reported the follow up (post placement) phase.

**Table 2:** Summary of included papers.

Author & year	Purpose of study	Participants	Timing	Setting	Methodology	Outcomes
(Abbey et al., 2006)	To investigate what aspects of clinical placements in aged care dissuaded Bachelor of Nursing students from considering a career in aged care and what could change this perception	Undergraduate nursing students who had completed clinical placements in residential care. Twelve Registered Nurses who acted as clinical teachers in the aged care facilities. n = 26	Follow up	Residential care	Qualitative research	All participants viewed aged care clinical placements as valuable opportunities to learn, practice, and master basic nursing skills. Clinical teachers and some students further emphasized that these placements allowed students to practice holistic nursing care. The clinical teachers highlighted the significant impact that registered nurses (RNs) with a strong understanding of aged care practices and networks can have. They highlighted the autonomy and diverse professional skills required of RNs in this field, which include managing staff, handling documentation for funding, providing care to residents, and offering counselling and support to staff, families, and residents. Many students expressed concern that the aged care setting did not provide opportunities to practice more advanced technical skills, which they considered central to modern nursing. Both students and clinical teachers raised issues about inadequate preparation for placements. Additionally, it was noted that the curriculum did not adequately prepare undergraduate students for aged care clinical placements. The experience of attending to naked bodies was highlighted as a challenge, particularly for both male and female undergraduate nurses. There was a need for students to be prepared for the emotional shock that may arise when encountering frail, damaged, or disfigured older bodies and minds. Some students associated their residential aged care placements with feelings of powerlessness and constraint, speculating that the lack of resources at both the site and industry levels affected the quality of care. Several participants also mentioned the challenges young RNs might face when working with long-serving care staff. Autonomy was seen as both a benefit and a burden, as nurses worked independently rather than being subordinate to medicine. Participants noted the low status and respect afforded to aged care nursing, attributing this to the sector’s ongoing funding and resource issues. Concerns about career progression in aged care were a significant factor in students’ decisions regarding future work in the field. Many saw aged care as a dead-end career pathway, with few opportunities for advancement or professional development. The lack of structured education opportunities and the high proportion of staff with low or no qualifications were also raised concerns.
(Alexander et al., 2025)	To understand the experiences and satisfaction of nursing students from a Victorian university with their mental health nursing placement.	Students undertaking Mental health nursing placement and enrolled in a Bachelor of Nursing or Bachelor of Nursing and Midwifery n = 264	Follow up	Mental Health Clinical Placement	Cross- Sectional study	Students consistently described positive clinical experiences when their preceptor or buddy nurse encouraged, facilitated, supervised, and supported learning opportunities that enabled them to achieve their placement learning objectives. Some students also described feeling welcome and valued during their clinical placement. Experiences of being treated with respect were commonly associated with positive clinical placement experiences. Many students reported not being given adequate opportunities to achieve their learning objectives during clinical placement. Some described isolating experiences where; despite actively seeking learning opportunities, they were denied the chance to participate in clinical activities. Several students felt unsupported, treated as a burden, and excluded from practising clinical skills, often being left in office areas while their buddy nurse engaged with consumers. Students frequently described experiences of incivility among staff within the clinical environment. Some perceived the workplace culture as cliquey and unprofessional, with nurses displaying hostile behaviours toward one another. Students also reported witnessing frequent arguments and undermining between clinicians, which at times resulted in them being discouraged from attending clinical review meetings due to the tense atmosphere.
(Alharbi, 2023)	To understand the perceptions of undergraduate nursing students working in the community and where they intend to pursue their careers	First to final year nursing students enrolled in a bachelor’s degree in three Saudi Arabian Universities n = 439	Follow Up	Community	Cross sectional study Questionnaire The Scale on Community Care Perceptions (SCOPE)	Ability to practice clinical nursing skills provided positive patient relationships. Students reported opportunities for advancement. The community setting was reported to be collaborative, with a positive occupational health work environment. Students reported a caregiving collaborative work environment with other disciplines. They had opportunity for contact with families. Prospect for freedom of action (autonomy) and professional development opportunities as a graduate were visible. Limited career advancement. Difficulty moving from community to acute care.
(Andrews et al., 2005)	To investigate how Bachelor of Nursing student work placement experiences impact workforce recruitment and what factors influence post graduate career choices	Undergraduate nursing students in 1st −3rd years in two universities and former students recently graduated within 12 months of survey n = 650	Follow Up	University	Mixed methods Questionnaire Focus Group Semi structured phone interviews	Placement provided a positive learning environment. It was a supportive environment. Positive facilitation and mentorship incidence experience. Positive staff interactions. Positive family interactions. Opportunity to be involved in care. Encouraged to give opinions, ask questions. Positive management. Respectful. Negative experience limited learning opportunities. Stressful environment. Frequent staff turnover resulted in no mentor/no support assigned. Allocated menial duties that did not facilitate learning (used as assistants in nursing, not students). Staff not wanting students- large numbers of students on one ward. Limited staffing putting pressure on students to work outside of their scope. Racism.
(Anyango et al., 2024b)	This study had three objectives: 1) to determine final-year nursing students’ career preferences and long- term career plans; 2) to investigate factors influencing nursing students’ specialty choices following their final- year clinical placement(s); and 3) to understand how final-year clinical placements can be used to develop the career interests of nursing students to different nursing specialties	Final year Bachelor of Nursing and Master of Nursing (Graduate Entry) students at two universities n = 555	Follow up	Hospital (75.1%) and non-hospital (24.9%)	Cross sectional Online survey Career Decision making Self-Efficacy Short Form b (CDMSE-SF)	Clinical environment. Positive staff attitudes. Supportive staff. Opportunities for patient interaction and diversity of work learning opportunities. Care complexity. Stress levels. Patient ratios. Team environment. Learning opportunities. Previous employment influenced experience and career goals. Remuneration for work was a positive outcome. Final clinical placement became preferred choice of speciality in graduate year. Preference to start career in acute care rather than aged care or mental health. Negative stereotypes and perceptions about specialties existed pre-placement. Poor clinical practices. Poor supervision. Inadequate staffing impacted students. Negative staff attitudes. Students felt unwelcome and ignored. Confusion on scope of practice by clinical supervisors. Staff fatigue influenced placement experience. Practice areas were associated with a concept of deskilling (mental health). Due to unsafe staffing levels, some staff were undertrained and resulted in staff burnout. Physical demands were high. Emotional demands of the job weighed on students. Lack of job opportunities was associated with perceptions of lack of job security.
(Anyango et al., 2024a)	This study examined the self-efficacy or confidence of final-year nursing students in making career decisions and the factors that influence their career decision-making process using an analytical cross sectional design.	Final year pre-registration nursing students in the Bachelor of Science (BScN) and the Master of Nursing (Graduate Entry) programs n = 222	Follow up	Acute Care	Cross- sectional study	Following dichotomisation of the CDMSE-SF scores, 171 students completed all questionnaire items. Of these, 47% (n = 81) achieved a score of 87.5 or higher, indicating good confidence in career decision-making, while 53% (n = 90) scored below 87.5, indicating lower confidence in career decision-making. Participants without previous nursing experience reported higher CDMSE confidence than those with prior nursing experience; however, this difference was not statistically significant (χ² = 3.265, df = 1, p = 0.071). Overall, the influence of previous nursing experience on decision-making varied across the subscales, with mixed results observed.
(Anyango et al., 2024a)	To explore the influence of clinical placements in the final year of nursing education on pre-registration student nurses’ career decisions	Final year nursing students from two Australian Universities n = 12	Follow up	Hospital settings (ICU, ED, medical & surgical wards) and non-hospital settings, (aged care & community)	Mixed Methods Semi structured interviews	Clinical environment. Variety and fast paced. Exposure to new areas. Positive client interaction. Achieved fulfillment in caring. Positive ward environment inclusive of teamwork. Innovative clinical placements (in the final year of nursing education) improve students’ learning and enhance career preferences in non-hospital settings. Interprofessional collaboration work opportunities were created. Positive management and leadership of ward lead to positive preceptorship. Clinical environment was repetitive, boring and mundane. There was a lack of physical nursing tasks in mental health settings. Understaffing and poor management resulted in lack of clinical facilitation and lack of learning opportunities. Students wanted to consolidate acute skills before specialising. Poor ratios and burnout in aged care settings with novice facilitators.
(Anyango et al., 2024b)	To 1) explore individual factors that impact the nursing specialty choices of final-year nursing students and 2) explore how these factors influence the final-year nursing students' career decision-making process.	Final year clinical placements of two nursing education programs, the Bachelor of Science (BSN) and the Master of Nursing (Graduate Entry) courses n = 12	Follow Up	Not reported	Qualitative	Some participants reported that their previous education exposed them to a range of healthcare issues and diverse client groups. Some students sought career advice from their clinical mentors and preceptors through self-initiated mentoring sessions during their clinical placements, which supported them in planning their future careers. Lecturers were also perceived as positive role models who fostered favourable attitudes towards specific client groups or specialties. International students reported feeling disadvantaged when beginning their careers compared with their domestic counterparts. Many indicated that they had limited choice when accepting graduate positions and felt more likely to accept placements in less preferred specialties than domestic nursing graduates. Students acknowledged the physical and emotional demands associated with their caring role. They described challenging experiences, including managing violent patients in mental health settings and caring for dying patients in palliative care wards. As a result, many indicated that they would be inclined to avoid certain clinical areas if they felt unable to cope with the demands of those environments.
(Bloomfield et al., 2015)	This paper reports findings from the first phase of a national study conducted to identify the intention of nursing students to work in a Primary Health Care (PHC) setting following registration.	Final year nursing students enrolled at Australian Universities n = 456	Follow up	Not reported	Case control study Online survey purposively developed	Learning about PHC in their curriculum. Support in PHC by facilitator to show variety of work available. Undertaking clinical placements in a PHC setting during their pre-registration education did not influence participants choice of PHC as preferred place of work following registration. Lack of career framework was a negative experience. Lack of PHC in the university curriculum resulted in lack of preparedness of graduates to work in PHC. There was a lack of structured and facilitated transition to professional practice programs in PHC environments.
(Boyd-Turner et al., 2016)	This paper explores how the student placement experience may influence employment choices in the context of paediatric nursing.	Newly qualified Bachelor of Nursing graduates who had qualified within previous 12 months and completed a workplace learning placement in paediatrics n = 12	Follow up	Tertiary teaching hospital which specialised in women, children and neonate health care delivery.	Qualitative research Semi structured interviews	Wish to work with children and job availability. Support available on placement. Assistance provided with career planning whilst on placement. Group debriefing for emotional support. Positive treatment by clinical staff whilst on placement including educational opportunities. Availability of clinical facilitation. Lack of supportive facilitation. Lack of education whilst on placement. Staff negativity toward students on placement with negative attitudes of staff observed due to workforce pressures.
(Brown et al., 2008)	To identify the role and influence of students’ learning experiences on shaping their predispositions to work with older people and to identify the characteristics of a positive learning environment that might promote a more favourable view of gerontological nursing	Student nurses in four schools of nursing across the years of training n = 718	Baseline and follow up	Not reported	Longitudinal mixed methods Focus group Placement visits Survey	Students do not enter nursing with negative perceptions of working with older people. Students who experience ‘enriched’ environments of care and learning, as characterised by the ‘Senses Framework’, can have their perceptions of gerontological nursing transformed so that they are more likely to view it as a positive career option. Prior positive experiences working with older people. Positive workplace learning experiences working with older people. Sense of belonging on placement. Good placement preparation. Welcoming environment. Support to achieve workplace learning goals. High standards of care. Feeling valued. Exposure to ‘impoverished’ environments of care during clinical placements or extra-curricular work can create negative perceptions of gerontological nursing. Negative student experiences on placement. Poor practice experienced on placements. Poor experiences on extra-curricular paid work. Experiences of poor patient care. Overworked, underappreciated, underpaid staff. Lack of resources and equipment. Lack of appropriately trained staff No staff training. Poor staffing levels. Lack of positive leadership. Limited learning opportunities on placement.
(Calma et al., 2022)	To explore final-year nursing students’ confidence, interest and intention to work in general practice	Final year nursing students from five Australian universities n = 355	Follow up	Not reported	Cross sectional study Descriptive purposefully designed online survey	Sufficient knowledge or skills to work in PHC. Preparedness of Bachelor of Nursing degree. Recent PHC workplace learning clinical placement experiences enable students’ socialisation to the setting and the role, which can ultimately shape career interest. The third main finding revealed that international nursing students were significantly more likely to be interested in seeking employment in general practice following graduation than domestic students. Given international participants in this study have come from different countries, the diversity in their own country’s health systems may have influenced their understanding of, and the value placed on PHC. Limited PHC in content in Bachelor of Nursing degree. Gaps in undergraduate nursing students’ knowledge, and inconsistencies in PHC content within an acute care focussed curricula contribute to nursing students’ low intention to seek PHC employment. Limited workplace learning opportunities in PHC.
(Cleary and Happell, 2005)	This paper reports the findings of a survey administered to undergraduate nursing students and trainee enrolled nurses within the mental health area. the purpose of the survey was to measure satisfaction with clinical placements within an area mental health service. it was intended that the findings provide direction for the on-going development and improvement of clinical placements, clinical supports, education programs and recruitment and retention initiatives within Central Sydney Area Mental Health Service.	Undergraduate Nursing Students and Trainee Enrolled Nurses on placement within an area mental health service n = 236	Follow Up	Inpatient and community services	Quantitative research Survey A nursing survey was constructed by the authors in consultation with key personnel	Preceptorship. Welcoming staff. Clinical experience in mental health. Education. No preceptor allocated. Nursing staff who seemed unwilling to have a student follow the/tag along for part of a shift. Unstructured levels of opportunity (e.g. some tasks being allowed one day but prohibited the next).
(Courtney et al., 2002)	To evaluate the effectiveness of a Clinical Placement Support Scheme for nursing students as a recruitment strategy for rural and remote health care services, and to develop an increased awareness of the employment opportunities available in these areas. The following research questions were explored: 1. Did the location of clinical placement have any impact on the intention to seek work in a rural area? 2. Which aspects of health care agencies encourage student nurses to seek future employment	Final year Bachelor of Nursing students at Queensland University of Technology n = 212	Baseline and follow up	Undertaking a rural placement in 3rd year.	Quasi-experimental pre-post-test survey The questionnaire, consisted of 38 items (16 Likert scale questions, 15 closed (yes/no) questions and 7 open-ended questions) was trialled on a similar group of students prior to distribution, and modifications made where necessary.	Friendly approachable staff, supportive learning environment. Good teamwork between staff. Preceptor support. Good staffing and facilities. Flexible working conditions available. Opportunities for career advancement No training support in rural areas. Desire to obtain clinical experience in large metropolitan hospital. Unfriendly, unsupportive staff. Poor staffing ratios and rostering. Unprofessional environment. Poor management. Very remote areas. Lack of diversity in choice of practice areas.
(Edmonds and Abdulmohdi, 2025)	To explore the placement experiences of final-year nursing students in one critical care setting, understand how these students coped during their final-year placement and the contribution this clinical placement had upon their learning as a final-year nursing student.	Final-year nursing students enrolled in the Bachelor of Science (BSc) program m = 6	Follow up	Critical Care	Qualitative research	Students felt empowered to identify their own learning needs and independently seek information to manage new caring situations. Students provided positive feedback regarding the support offered by the teaching team. This support assisted nursing students in developing a clearer understanding of their activities and the underlying rationale for them. Fifty percent of students reported that they had been prevented from participating in certain experiences due to their student status. This restriction was perceived negatively, with participants expressing frustration that their learning opportunities had been constrained as a result. Nursing students were required to prepare for a level of knowledge that exceeded their initial expectations. While they acknowledged the associated benefits, the elevated expectations contributed to feelings of anxiety and perceived inadequacy.
(Foster et al., 2021)	To explore nursing students’ experience of traditional mental health clinical placement and how it influenced their practice and their understandings of recovery from mental illness.	Undergraduate nursing students at a metropolitan health service n = 31	Follow Up	Large Australian metropolitan public mental health service	Qualitative research Four semi structured focus groups	Mental health placements are crucial in attracting students to the field and should remain an integral part of comprehensive pre-registration education. These placements offer students valuable opportunities to build rapport with consumers through everyday activities and conversations, allowing them to see individuals beyond their diagnoses or labels. Such experiences help students recognize and challenge their own personal biases, negative assumptions, and stigmatizing attitudes toward mental illness and those affected by it. The placements foster an inclusive, multi-disciplinary team environment where positive interactions between staff and clients are encouraged. Students also experience inclusivity in patient care planning and are supported by collaborative nursing teams. The supportive atmosphere, along with a positive work environment, allows students to develop the interpersonal and empathetic skills necessary for mental health nursing. Overall, the placements nurture a positive team culture, enhancing both professional and personal growth. The stigma surrounding mental health as an illness can impact students' willingness to pursue it as a career path. Some students expressed apprehension before their placements, fearing they would be exposed to frightening behaviours and violence from consumers. Issues such as poor preceptorship, being overlooked, and having limited opportunities to engage in nursing tasks due to extended periods of downtime also negatively affected the experience. Additionally, students were influenced by traumatic client stories and negative staff behaviour, which contributed to a sense of workplace incivility. Students also experienced vicarious trauma from witnessing the distress of mental health consumers and hearing their personal stories, which further impacted their perceptions of the field.
(Gale et al., 2016)	This article presents the outcomes of a pilot project that was undertaken in one university in London in collaboration with several general practitioner (GP) practices local to that university. This collaboration involved student nurses from one cohort at the university undertaking placements with practice nurses where the GP employs practice nurses. The evaluation also explored the impact on student learning and the value that the placement had.	Undergraduate nursing students who undertook clinical placement in a GP practice n = 17	Follow Up	GP practice	Mixed methods Survey	High quality mentorship. Welcoming environment. Autonomous work environment. Worked alongside doctors rather than being told what to do. Inter-professional environment. Strong teamwork. Improved communication skills Preparation programme. The only negative response identified from the data was that the placement should have been longer, placement 6–8 weeks.
(Happell, 2008)	The focus of this paper is on participants stated intention to apply for a graduate program and to pursue a career in psychiatric/mental health nursing. These factors are considered in light of perceived preparedness for mental health practice, encouragement to consider a career in this field and the provision of career information.	Bachelor of Nursing students in Victoria n = 783/688	Baseline and follow up	Mental health placement,	Pre and post Test Questionnaire Psychiatric / Mental Health Clinical Placement Survey	Clinical placement in mental health enhances desirability to choose it as a graduate specialty as they feel more prepared. Career advice encouraged by other staff. Lack of preparation.
(Hunter et al., 2015)	To describe Masters entry nursing students’ attitudes about psychiatric mental health clinical experiences; preparedness to care for persons with mental illness; students’ perceived stigmas and stereotypes; and plans to choose mental health nursing as a career.	Graduate entry nursing programme in USA n = 30	Baseline	Masters’ entry, pre-licensure graduate nursing students University in a large city in the Midwestern US.	Quantitative descriptive design Self-administered paper and pencil Nursing Students’ Attitudes about Psychiatric Mental Health Nursing questionnaire	Supportive staff. Increased amount of clinical time. Active involvement in patient care. Faculty support. Preparedness for clinical placement. Emphasis of mental health nursing importance in curriculum. Uncertainty and anxiety about caring for people with mental illness. Negative stereotypes of mental illness.
(Kloster et al., 2007)	This paper is a report of a study to identify Norwegian undergraduate nursing students’ career preferences at the beginning and end of their nursing education programme, together with their reasons for these preferences.	Undergraduate nursing students in five nursing schools phase 1 n = 620 phase 2 n = 386	Baseline and follow up	University	Longitudinal descriptive design Questionnaire with closed and open-ended questions	A significant portion of participants, 83.7%, agreed that their clinical practice experiences influenced their choice of work area. Additionally, 79.0% of participants noted that the primary reason for choosing a work area was the nursing challenges offered. Changes in preferences between Phase 1 and Phase 2 were largely attributed to experiences during clinical placements, personal growth, and a growing understanding of the nursing profession. Those who expressed interest in working with older people mentioned that the work was meaningful to them, as they enjoyed spending time with older individuals and felt a sense of responsibility in addressing the challenges in aged care. Participants interested in home-based care valued the work for being self-directed, challenging, and meaningful. On the other hand, those considering mental health nursing were drawn to the exciting and challenging environment, where they could apply their personal competencies and experiences, and appreciated the fact that the job did not involve heavy physical tasks. Boring work. Physically and psychologically demanding work. Untrained staff. Too busy with insufficient time to finish work. Poor leadership. Poor work conditions. Psychological stress.
(Lea et al., 2016)	To investigate which aspects of student nurses’ experiences of residential aged care facility clinical placements affect perceived likelihood of choosing a career in residential aged care post-graduation.	2nd year Bachelor of Nursing students in Tasmania n = 71	Baseline and Follow Up	Residential Aged Care	Quantitative Action research mentor group Pre- and post-placement student surveys containing five-point Likert-type scales for each response options.	Opportunity to work with elderly people. Ability to provide continuity of care. Supportive mentors. Other staff with a positive attitude. Teaching and learning environment. Opportunity for regular two-way feedback. Good preparation for placement. Low professional status of aged care.
(Lea et al., 2008)	To investigate whether rural clinical placements for student nurses at a rural university in New South Wales influence their decision to join the rural and remote registered nurse workforce.	Final year Bachelor of Nursing students at an NSW rural University n = 75	Baseline and Follow Up	Rural health services	Qualitative research Descriptive survey design Two surveys which consisted of closed (yes/no) and open-ended questions Individual in-depth interviews	Learning experiences available. Positive culture. Student rural origins. Financial considerations. Variety of clinical experiences. Opportunity to practice skills. Supportive team environment. Relaxed, friendly environment. Close to home (rural students). Supportive staff. Knowledgeable staff. Lack of resources and technology. Lack of support available for permanent staff working in rural patient acuity. Skill mix. Demanding workloads. Lack of support for graduate program. Limited availability of clinical rotations such as intensive care unit and emergency departments.
(Levett-Jones et al., 2007)	This paper reports on some of the findings from the qualitative phase of a mixed-method, multi-site study that explored nursing students’ experience of belongingness while on clinical placements.	3rd year Bachelor of Nursing students from three universities in Australia and UK n = 18	Follow up	One Mental health Two – not reported	Qualitative research Semi-structured in-depth interviews	Welcoming, accepting and supportive leaders and facilitators. Inclusive and encouraging staff. Quality mentorship. Alienation from unreceptive environments.
(Lim et al., 2020)	This study examined the relationship between clinical placement, confidence and stigma surrounding mental illness. How do nursing undergraduates' (i) attitudes toward people with mental illness (ii) perceptions of clinical confidence in performing essential mental health nursing skills, and (iii) attitudes toward their mental health clinical placement experiences affect their intention to work in mental health settings.	3rd /4th year Bachelor of Nursing students n = 144	Follow Up	Mental health facilities	Correlational design Questionnaires The 22-item Clinical Placement Survey (CPS) scale 20-item Clinical Confidence Scale (CCS) 7-item Social Distance Scale (SDS)	Nursing undergraduates' attitudes toward their mental health clinical placement experiences were found to have a moderate, significant correlation with their clinical confidence. Higher perceived clinical confidence was notably linked to a greater sense of preparedness, reduced anxiety toward individuals with mental illness, and the belief that their nursing curriculum had equipped them for various nursing specialties. The strongest predictor of nursing students' likelihood to choose a mental health setting as a future career was their willingness to engage with individuals experiencing mental health conditions. Overall, students expressed a positive attitude toward their mental health clinical placements, as these experiences helped prepare them for mental health nursing, enhanced their understanding of mental illness, and reduced their anxiety about working with individuals facing mental health challenges. Staff stress. Heavy workloads. Poor staffing.
(McCall et al., 2009)	To explore the influence that clinical placements have on career intentions for undergraduate midwifery student.	Bachelor of Midwifery students who had either undertaken their first or last clinical placement n = 11	Follow Up	Hospital and community maternity services	Qualitative research Focus groups and individual interviews (telephone)	Clientele reality of the profession "opened eyes" to role. Opportunity to gain experience and confidence in an area as a new graduate. Placement provided opportunity to secure graduate work. Positive experiences preferred choice for post graduate work. Students whose expectations did not align with their experiences, or who had challenging placements, considered deferring or even withdrawing from the course. Some expressed concerns that relocating to rural areas may reduce the amount of instrumental and occupational support they could receive. While clinical placements were seen as an opportunity to secure future employment, this was challenging for students who did not wish to work with the organization where they completed their placements. Additionally, some students who had declined job offers from their placement organizations noted that this created tension and strain in relationship with their supervisor. Rural clinical practice was also seen as unfeasible by some students, particularly those who believed rural hospitals would be unlikely to hire newly graduated midwives.
(McKenna et al., 2010)	To explore the influence of health professional students’ clinical placements on their future career intention	Bachelor of Nursing students who had completed their 1st or 3rd year clinical placement n = 13	Follow up	Clinical placement type not reported	Qualitative Focus groups n = 3 Individual interviews n = 10	Clinical placements offer exposure to a variety of settings. Range of activities - interesting work diversity. Opportunity to gain experience and learn. Close to home/family. Supportive staff. Poor quality clinical placement. Stress in environments. Isolation of rural setting.
(Neill and Taylor, 2002)	In this article two arguments are advanced: (i) providing rural and remote clinical experiences for under- graduate nursing students, especially those from urban backgrounds, offers recruitment possibilities; and (ii) adequate financial support for students during these placements is essential	Bachelor of Nursing students Flinders University n = 33	Follow up	Hospital, community and remote health centres of central Australia, Eyre Peninsula and Northern South Australia	Qualitative	Given the right encouragement, support and preparation, students demonstrated they quickly become valued contributors and culturally safe team members. Students found their experiences in rural and remote venues changed their lives and future careers. Made to feel as part of the healthcare team. Friendliness and open acceptance by the community. Funding issues working remotely.
(O'Brien et al., 2008)	To evaluate the effectiveness of using clinically based facilitators for undergraduate nursing students on mental health placements. To increase the knowledge base of all registered nursing staff of the needs of undergraduate students on clinical placement. To support individual units to develop clinical placement packages that can be used as a continuous resource. To increase the collaboration between mental health services and a university nursing program.	2nd and 3rd year Bachelor of Nursing students undertaking a placement in mental health 2nd year n = 254 3rd year n = 248	Baseline Follow up	Mental health facility	Mixed methods Pilot study Questionnaire x 3 pre-placement post- placement, and placement evaluation	Facilitators. Involvement by staff. Interaction with patients. Prior concepts of mental health placements
(Patterson et al., 2018)	To investigate the impact of nursing student participation in a non-traditional mental health placement, Recovery Camp (RC), compared to participation in traditional placement	3rd year Bachelor of Nursing students undertaking traditional and non-traditional mental health placements n = 50 Intervention group n = 23 Comparison group n = 27	Baseline Follow up	Traditional mental health placement versus Non - traditional mental health placement- Recovery Camp	Cohort Study The Preplacement Survey	Increased preparedness for mental health nursing post graduating from RC placement. Decrease in anxiety of dealing with MH after RC. Increased clinical confidence post RC. No significant differences between non-traditional and traditional placements were observed in perceived Course effectiveness. No significant difference was found between the groups in the subscale "Future Career".
(Peters et al., 2015)	To explore the experiences of pre-registration nursing students following community based clinical placements and to explore the impact of this placement on their learning.	Graduate entry and combined degree Master of Nursing Students n = 24	Follow up	Community based clinical placement	Qualitative In-depth semi-structured interviews	Holistic focus of community nursing. Autonomy. Independence. High level of expertise. Nurse actively involved students. Increased student learning. Teamwork. Slower pace of work enabled learning. Perception of need for advanced skills may negatively impact student choice.
(Salamonson et al., 2018)	To explore students’ perceptions of their clinical placement experience in a community-based alcohol and other drugs (AOD) rehabilitation setting, and to examine attitude career intention, and satisfaction with the placement.	Undergraduate health students from three health-related programmes (nursing, medicine, and counselling) in the Sydney metropolitan region (NSW, Australia). n = 58	Baseline Follow up	clinical placement at a community AOD rehabilitation centre.	Mixed Methods Pre-post-test surveys Attitude to Recovery-Oriented Practice-13 scale (ARP-13) Subjective Norms, Perceived Behavioural Control, and Intention to Pursue a Career in AOD Rehabilitation Setting (ASPIRE-ADR) Clinical Learning Environment Inventory-19 (CLEI-19) scale Semi structured interviews	High satisfaction with clinical placement experience. Improved knowledge and skills. Positive client interaction. Different setting from a hospital. Positive interaction with staff - felt part of a team. Enthusiastic caring staff. No new skills learnt. Limited work for a student nurse. Unprepared for placement due to lack of material in curriculum. Confronting at times due to client type. Lack of placement structure limited facilitation.
(Thongpriwan et al., 2015)	To describe undergraduate nursing students’ attitudes toward mental health nursing and how these attitudes influenced their professional career choices in mental health nursing.	Undergraduate nursing students at the junior and senior level (or equivalent) enrolled in either an Associate Degree in Nursing (ADN) or Bachelor of Science in Nursing (BSN) program in a Midwestern state of the U.S n = 229	Baseline	University	Cross Sectional Survey Modified scale - the Mental Health Nursing Student Survey	Preparation prior to placement. Positive placement experiences. There were no significant differences in preparedness between students based on age, gender, or interest in a summer externship in mental health (MH) nursing. Factors such as age, gender, ethnicity, nursing level, nursing program, and prior experience or educational preparation in MH nursing did not significantly affect students' knowledge or attitudes toward mental illness. Students with no experience (either in class or clinical settings) demonstrated significantly more negative stereotypes about mental illness compared to those who had experience. However, no significant differences in negative stereotypes were observed between students of different ages, genders, ethnicities, nursing programs, nursing levels, or MH experience. Additionally, no significant differences were found in students' interest in a future career in MH nursing based on these factors. Students expressing higher anxiety about mental illness were typically those in Bachelor programs, with no experience or only non-nursing roles in MH, no coursework, or no clinical practice in MH settings. Anxiety levels did not significantly vary by age, gender, ethnicity, nursing program, or interest in an externship.
(Wareing et al., 2018)	This study sought to answer the following research questions What impact do clinical placements have on pre-registration nursing students’ choice of clinical specialty? What factors do pre-registration nursing students perceive as important in terms of the desirability of a clinical specialty? At what stage do clinical placements begin to influence the employment intentions of pre-registration nursing students?	Final week of the Bachelor of Science (Hons) nursing programme n = 75	Follow Up	Two university campus locations	Cross Sectional Survey - Mixture of Likert scale and free-text responses from pilot study	Sense of belonging. Interpersonal relationships with staff. Learning experience, wide range and new knowledge gained. Quality of working environment. Teamwork felt included. Positive leadership. Positive patient experiences. Ability to provide one on one care work independently. Mentors. Level of support. The range of placements that had been on offer by the university. The students preconceived ideas.
(Wareing et al., 2017)	To explore the influence of practice placements on final-year adult nursing students’ career decisions	Final year nursing students n = 35	Follow Up	Two university campus locations	Mixed Methods Cross Sectional Questionnaire – study specific	Support offered by mentors and clinical staff. Opportunity to make a difference to patients. Poor staffing levels. Lack of support.
(Williamson et al., 2023)	To implement and evaluate Collaborative Learning in Practice (CLIP) in General Practice (GP) nursing settings To explore issues of interprofessional learning GP settings To explore patient access to services related to CLIP and increased student nurse capacity in GP practices	Student nurses n = 31	Follow Up	Six GP clinics within local primary care networks	Mixed Method Focus groups Patient access audit	Welcoming staff. Sense of belonging. Peer support. Interprofessional learning opportunities. Autonomy to practice. Enhanced responsibilities. Increased capacity of clinic appointments. Not reported.

### 4.1 Surveys

Seventeen studies in this systematic review used surveys to collect data. No two studies used the same questionnaire therefore it was not possible to combine results. A wide range of standardised and study specific surveys were used (See Table 2). Study specific surveys were designed based on previous studies specific to the organisation undertaking the research.

Anyango et al. (2024c) reported 47% of final-year students (n = 81) achieved a CDMSE-SF score ≥ 87.5, indicating high confidence in career decision-making, while 53% (n = 90) scored below this threshold. Previous nursing experience did not significantly influence confidence (χ² = 3.265, df = 1, p = 0.071). Similarly, Hunter et al. (2015) found that supportive staff, increased clinical time, and faculty involvement improved preparedness for mental health placements, although students also reported uncertainty and anxiety prior to placement. Cleary and Happell (2005) reported that students valued preceptorship, welcoming staff, and structured learning opportunities. However, 236 students also identified inconsistent access to clinical tasks, including days where certain activities were permitted and others where they were prohibited. Alexander et al. (2025) found that although many of the 264 surveyed students described positive experiences when supported by preceptors, a substantial proportion reported limited opportunities to meet learning objectives, experiences of exclusion, and workplace incivility. However, the findings from the surveys and subsequent analysis yields results which closely align to the qualitative studies included in this review.

Integration of quantitative and qualitative findings across the included studies demonstrates a coherent and mutually reinforcing relationship between clinical placement experiences, undergraduate nursing students’ perceptions of mental health nursing, and their subsequent postgraduate career preferences. While quantitative studies provided measurable indicators of satisfaction, confidence, and career intention, qualitative evidence highlighted the interpersonal, organisational, and contextual factors that shaped these outcomes. Data relevant to qualitative finings was extracted to gain a deeper understanding of nursing students experiences and perceptions of their clinical placements. Through this process the research team were able to identify themes and subthemes across studies increasing the understanding of nursing students’ experiences and conduct a synthesis of the results informed by the students’ perspectives.

### 4.2 Key themes

Key themes which emerged from this systematic review were ***empowerment through supportive clinical engagement*** through or autonomy and confidence, opportunities for hands on clinical skills and positive mentoring, ***barriers to professional growth in clinical practice*** included lack of support and training, limited learning opportunities, feeling unwelcomed and unchallenging work activities. Finally ***practice area type***, mental health, critical care and aged care were associated with negative clinical placements experiences however general practice, intensive care units, and dedicated university training modules for specific clinical placement types were positive contributing factors for clinical practice experiences. The themes found from the studies align with a recent scoping review (Wilson et al., 2026) which mapped known experiences of students on mental health placements internationally and a systematic review which explored the available evidence for care homes as a clinical placement domain for nursing students (Adeboye et al., 2025) .

#### 4.2.1 Empowerment through supportive clinical engagement

Empowerment through supportive clinical engagement centred around opportunities for autonomy and professional advancement, as well as the development of hands-on clinical skills. This was found in qualitative and mixed methods studies, **Autonomy in practice** was frequently identified as a key factor in fostering a sense of competence and confidence among nursing students and this was found across multiple clinical practice areas (Abbey et al., 2006; Alharbi, 2023; Gale et al., 2016; Peters et al., 2015). During placements in community health and primary care settings, students reflected that nursing practice in these environments involved greater autonomy compared to that in the acute care or critical care setting:

*You make your own decisions to achieve and to see the patient being happy at the end of the day and being able to manage the illness — the chronic illness — and to be able to see the positive result at the end of the day. Or like when I went for an acute care placement. It wasn’t like that.* (Peters et al., 2015, p. 178)

In these environments, nursing students were given greater responsibility in decision-making and patient care, which in turn contributed to their professional growth and readiness for independent practice. Engaging with mental health nurses expanded students understanding of the profession, as they observed that mental health nurses exercised a significant degree of autonomy in their practice.

*Mental health nurses, they’re not just there to give depots ([sic]injection) or to just give medication. There’re other roles that mental health nurses do that general nurses wouldn’t do.* (Foster et al., 2021, p. 88)

Courtney et al. (2002) reported pre- and post-placement changes among 212 final-year students undertaking rural placements. Positive influences included friendly staff, supportive teamwork, good facilities, and preceptor support. Negative influences included poor staffing ratios, unsupportive environments, and limited diversity of practice areas. These factors shaped students’ intention to seek rural employment.

Additionally, the acquisition of hands-on clinical skills was emphasized as a crucial aspect of learning, with students noting the importance of **direct, practical experience** in developing the technical competencies required for post graduate careers, students were given greater responsibility in decision-making and patient care, which in turn contributed to their professional growth and readiness for independent practice.

*The staff knew it was our final placement and let us work independently. We were assigned a three-patient load, and I planned the patients' care and did their medications and ADLs without being interrupted. The nurses were approachable and always willing to teach us. The autonomy and good supervision benefited me; my confidence and knowledge grew significantly. I feel ready for the RN role.* (Anyango et al., 2024a, p. 73); Courtney et al. (2002)

Through this hands-on practice students on clinical placement in care homes has a greater understanding of person- centred care gaining hand on skills while working with diverse populations.

Welcoming and well-resourced work environments had a positive effect on students’ clinical placement experiences. Placement locations with appropriate staffing, sufficient resources and technology, high standards of care and a **culture of inclusivity** were viewed highly by students. This aspect was described as an 'enriched' environment by Brown et al. (2008) but featured in many studies. Cleary and Happell (2005) also reported evidence of this is their quantitative surveys across inpatient and community mental health placements.

*Because they [staff] were happy at work, they were better with the patients. You are going into work happy, the staff were all great, having a laugh, getting on with the job, and you had happy patients.* (Brown et al., 2008, p. 1226)

Equally important were the **empowering mentoring** relationships reported by the students. Empowered mentoring was associated with increased support, guidance, and role modelling, which helped foster professional identity and provided emotional and educational support. Empowering mentoring was noted for its ability to bridge the gap between theoretical knowledge and clinical application, offering students a sense of connection and direction within the clinical setting. A collaborative, multidisciplinary clinical environment was viewed favourably by students. This was also a finding by an earlier study by Courtney et al. (2002) which highlights that this is a core element to clinical placements for nursing students which has been reported for a significant duration of time.

*It is the experience that you have when you are on placement that can make the trusts more appealing. If you have a bad experience on placement you are less likely to want to go back there.* (Andrews et al., 2005, p. 147)

Remuneration for clinical placement work and other **financial supplements** were viewed positively by students. Financial support was particularly valued by students undertaking placements in rural locations. This assistance was appreciated in offloading pressures associated with being isolated geographically (Lea et al., 2008).

*My placements were costly however, in terms of lost wages from part-time work, and in living away-from-home expenses. Without some help from the University, I would have been unable to take these placements at all.* (Neill and Taylor, 2002, p. 242)

Happell (2008) demonstrated that mental health clinical placement increased students’ intention to pursue mental health nursing as a graduate specialty, with improved perceived preparedness following placement. In contrast, (Bloomfield et al., 2015) found that undertaking a primary health care placement did not increase intention to work in primary health care post-graduation among 456 final-year students. Calma et al. (2022) similarly reported low intention to seek primary health care employment, with international students significantly more likely than domestic students to express interest in general practice roles.

#### 4.2.2 Barriers to professional growth in clinical practice

Conversely, barriers to professional growth in clinical practice were also prevalent in the included studies. A **lack of support** and inadequate training were frequently cited as significant barriers to effective learning and professional development. Nursing students reported feeling underprepared for the demands of clinical practice, which was often exacerbated by insufficient guidance or structured educational frameworks. This was evident in both qualitative (Anyango et al., 2024a) and cross sectional studies (Alexander et al., 2025) with higher reporting rates by international nursing students. This lack of curriculum preparedness and faculty support undermined the potential for skill acquisition and diminished the overall educational value of the placement (Calma et al., 2022).

*“I felt I didn’t know anything – no idea of the structure around drug and alcohol rehab, as there was nothing taught at uni*.”(Salamonson et al., 2018, p. 799)

Equally, a lack of support from both placement mentors and clinical staff was frequently cited as a significant barrier to effective learning, professional development, and a sense of belonging while on clinical placement. Work environments that were under-resourced or unwelcoming had a detrimental effect on health students' experiences during their placement.

*About half of the nurses had resigned two weeks before my final placement, and nurses were dragged in from other wards and didn't want to be there. So, that led to huge tensions… My summation of the problem is that there was an issue with the nurse manager, the ward culture and certain payment systems… The placement was challenging…*(Anyango et al., 2024a, p. 73)

Furthermore, **limited learning opportunities** and the presence of unchallenging work activities was associated with negative clinical experiences becoming barriers to professional growth in clinical practice. In some instances, students found themselves performing tasks that were perceived as repetitive or menial, which did not align with their educational objectives or career aspirations. This lack of engagement with complex or meaningful tasks resulted in dissatisfaction and limited professional growth.

“*It is not very pleasant being used as a HCA (health care assistant) when you are supposed to be a student nurse, trying to gain the various clinical skills required to become a registered nurse”* (Andrews et al., 2005, p. 148)

### 4.3 Practice area type

The practice area type played a significant role in shaping the clinical experience. Mental health, critical care and aged care placements were associated with negative experiences. These areas were often perceived as challenging, which contributed to feelings of frustration when students were not adequately supported. Nursing student reported that some cases they were prevented from participating in certain care events and experiences due to their student status – this increased their dissatisfaction (Edmonds and Abdulmohdi, 2025).

*“it was emotionally hard to get used to it — you are dealing with yucky, horrible stuff all the time and [staff]all don't care”.* (Abbey et al., 2006, p. 16)

In the area of mental health, students reported encountering difficulties related to the stigma and challenges associated with mental health disorders, which resulted in a sense of alienation or disillusionment.

*Working with the homeless [team], it made me reflect on what it would be like to be homeless and not have anyone to call for help… So after my day I would write it down, pretending I was in that position.* (Foster et al., 2021, p. 88)

Similarly, aged care settings were often characterized by limited opportunities for skill development, leading to feelings of underutilization and reduced professional satisfaction.

*“It doesn't prepare us for acute nursing responsibilities ie. drips, arrests etc.”* (Abbey et al., 2006, p. 16)

However, some practice area types provided the opposite (positive) experience. Positive placement preparation and mentorship increased perceived likelihood of choosing aged care, whereas negative experiences reduced interest. This was noted in the primary health care settings and by international student, where nurses were viewed as valued professionals in the patient care team. This is in contrast to Anyango et al. (2024d) qualitative study where international nursing student felt more disadvantaged compared to their domestic student colleagues.

*“I didn't realise (there) can be specialist nurses or medicines prescribers at GP surgeries. Also, they were autonomous with their patients”* (Gale et al., 2016, p. 231)

Good **placement preparation** through appropriate curriculum content, as well as adequate faculty support, gave students confidence to practice their clinical skills while on placement.

*I found one of the tutors used to use practical examples and he really made it very interesting. He put theory and practice together… the academic [work] has been very helpful in helping me to understand how I feel about nursing.* (Brown et al., 2008, p. 1224)

These findings were reported across multiple geographical regions and across all nursing practice domains (Alexander et al., 2025; Hunter et al., 2015; Wareing et al., 2017)

Placement experiences provided nursing students the opportunity to **gain career advice** from their mentors and other clinical staff. In weighing up career pathways, students frequently considered potential for career growth, opportunities for advancement, availability or lack of job prospects, and workplace learning opportunities.

*“I spoke to the RNs I worked with for advice on what to do for a long-term career in ED.”* ( p. 74)

Long term post graduate career choices were also influenced by life experiences (Anyango et al., 2024a) found 47% of participants in their study had good confidence in career decision making. The authors acknowledged that pervious nursing experience was not statistically different p = 0.071 across their groups.

## 5 Discussion

This mixed-methods systematic review demonstrates that the quality of clinical placement experiences—rather than the placement setting itself—is the strongest influence on nursing students’ perceived post-graduate career preferences. Across quantitative and qualitative studies, supportive staff, structured learning opportunities, autonomy, and positive mentoring consistently increased interest in a specialty, whereas poor supervision, limited learning opportunities, and negative workplace cultures discouraged students from pursuing work in those areas. These findings align with international literature showing that placement quality is a critical determinant of workforce attraction and retention (Adeboye et al., 2025; Wilson et al., 2026). This systematic review synthesised evidence from 35 studies across multiple practice settings, on understanding the impact of clinical placements on nursing students and the influence of these placements on their perceived post graduate career preferences.

Clinical placements in Australia play a critical role in the development of nursing students, providing them with valuable hands-on experience in unique healthcare environments (Tran et al., 2024; Wiggins et al., 2022). These placements, while offering a range of positive opportunities for professional and personal growth, also present challenges that can affect students' perceptions of fields of practice and influence their career preferences (Tamata and Mohammadnezhad, 2023). This aligns with the findings of this systematic review where empowerment through supportive clinical engagement positive experiences, barriers to professional growth in clinical practice and practice area types were three key themes identified.

Supportive clinical environments consistently empowered students by fostering autonomy, confidence, and professional identity. Across multiple studies, students described how positive mentoring, constructive feedback, and opportunities to independently manage patient care enhanced their readiness for practice. One of the key benefits of nursing placements in community settings and rural areas was the opportunity for nursing students to gain exposure to a diverse range of cases, gaining breadth of experiences not always available in metropolitan settings (Alharbi, 2023; Lea et al., 2008). Hands-on clinical skill development was another key contributor to empowerment. Students who were trusted to manage patient loads, administer medications, or plan care reported significant increases in confidence and preparedness for the nursing role. In these settings the workforce is often smaller, resulting in nursing students gaining opportunities to work closely with multi-disciplinary teams, facilitate collaborative care and enhance their own learnings (Adeboye et al., 2025; Lea et al., 2008). These diverse environments foster professional growth, as students are required to take on a range of tasks, from clinical care to administrative duties, offering more comprehensive skills that would be harder to achieve in larger, more specialized metropolitan hospitals (Edmonds and Abdulmohdi, 2025).

Nursing students on these placements in community settings and rural areas developed stronger interpersonal skills due to the close-knit nature of rural communities (Courtney et al., 2002; Neill and Taylor, 2002). The opportunities to build meaningful relationships with patients, families, and healthcare teams was a vital aspect of these placements, particularly in aged care, where holistic and person-centred care was essential (Abbey et al., 2006). The challenges faced by nursing students in rural placements, such as limited resources caused frustration and distress (Andrews et al., 2005) however when mentorship is used as a training model during these placement types this can cultivate resilience, critical thinking, and autonomy in students (Anyango et al., 2024a).

Autonomy was particularly valued in community, primary health care, and mental health settings, where students observed nurses exercising independent decision-making and holistic care (Abbey et al., 2006; Alharbi, 2023; Gale et al., 2016; Peters et al., 2015). These experiences helped students recognise the breadth of nursing roles beyond acute care and contributed to increased interest in these specialties.

Student who received placement preparation for specific placement types experienced a reduction in stigma surrounding mental health and rural placement prospects (Bloomfield et al., 2015). The placement preparation supported students’ confidence regarding personal interactions with individuals suffering from mental illness (Patterson et al., 2018; Williamson et al., 2023). These placement preparation curriculums can lead to a reduction in preconceived biases and anxieties surrounding mental health care, thus improving students' attitudes and preferences toward these practice area types (Patterson et al., 2018).

Despite the benefits, clinical placements also come with distinct challenges that present barriers and can negatively affect students' learning experiences and long-term practice preferences. One of the primary concerns in this systematic review was the lack of sufficient support and resources in mental health and aged care settings (Brown et al., 2008). Many services were understaffed, and students found themselves overwhelmed by the workloads and experienced limited opportunities to engage in clinical activities (Anyango et al., 2024b). The shortage of experienced mentors or preceptors in these settings can hinder students' development, as they may not receive the structured guidance necessary for skill acquisition and confidence building (Adams et al., 2021; Jirwe and Rudman, 2012).

Additionally, nursing student on rural placements felt isolated due to the physical distance from metropolitan centres and lack of social support networks .(Alexander et al., 2025; McKenna et al., 2010) Relocating to a rural area for clinical placement exacerbated this sense of isolation, particularly for students who were unfamiliar with the rural lifestyle. For some students, the lack of access to professional development opportunities, and absence of career pathways also influenced their post-graduation preferences which is a known concern for existing workforce in these regions (Anyango et al., 2024a). In some mental health settings, additional challenges existed due to the limited availability of specialized mental health services and professionals (Alexander et al., 2025; Foster et al., 2021). Nursing students were exposed to vicarious trauma, as they witnessed the personal struggles of individuals (Foster et al., 2021). The emotional toll of working in such environments can lead to burnout and compassion fatigue, particularly for individuals who are in the early stages of their careers (Anyango et al., 2024a). These findings align with Wilson et al. (2026), who noted that stigma and lack of preparation contribute to student apprehension in mental health placements.

The findings of this review have important implications for nursing education and workforce planning in Australia. Given that mental health, aged care, and primary health care are core components of undergraduate nursing curricula, improving placement quality in these areas is essential for addressing workforce shortages. Strengthening preceptor training, ensuring adequate staffing, and embedding structured learning opportunities may enhance student experiences and increase interest in high-need specialties. Curriculum reform is also needed to better prepare students for placements in mental health, aged care, and community settings. Enhanced theoretical preparation, simulation-based learning, and targeted pre-placement modules may reduce stigma, increase confidence, and improve readiness for practice. From a workforce perspective, clinical placements should be recognised as a recruitment strategy. Positive placement experiences can attract students to specialties facing shortages, while negative experiences may deter them. Investment in placement quality—particularly in rural and remote areas—may support long-term workforce sustainability.

However, several limitations must be acknowledged. Database coverage was limited to MEDLINE and CINAHL, which may have resulted in relevant studies being missed. Although these databases are the most comprehensive and authoritative sources for nursing, and clinical education research, relying solely on two databases introduces an inherent risk of publication bias and incomplete retrieval of the global evidence base. Important studies indexed in other repositories may not have been captured. Additionally, limiting the search to English‑language publications may have excluded relevant international studies, particularly from non‑English‑speaking regions where nursing education and clinical placement structures differ. The heterogeneity of study designs and survey tools limited opportunities for meta-analysis. Many studies relied on self-report data, which may introduce bias. Sample sizes varied widely, and some practice areas (e.g., mental health) were underrepresented. Finally, most studies were conducted in high-income countries, limiting generalisability to other contexts.

## 6 Conclusion

This systematic review provides an overview and an understanding of the impact of clinical placements experiences on nursing students and the influence of these placements on their perceived post graduate career preferences. The empowering influences included exposure to diverse cases, the development of close professional relationships, and the cultivation of flexibility and autonomy. However, the lack of resources, support, and professional development in some settings can hinder or present barriers for students’ learning experiences. There is a risk social and professional isolation may impact nursing students' well-being. While mental health and aged care placements in rural and remote Australia offer significant educational opportunities, these present certain challenges. The stigma associated with mental health further complicates learning experiences for students preparing for placement. The inclusion of specific practice type information in curriculums, the use of preceptorship opportunities during placements and exploration of non-traditional placement types could address these challenges to ensure emerging graduate nurses are adequately supported to foster a sustainable nursing workforce in the future. Further research is required in these domains to fully explore and understand their benefits and considerations for implementation to improve placement experiences.

## CRediT authorship contribution statement

**Kristina Griffin:** Writing – review & editing, Writing – original draft, Project administration, Methodology, Investigation, Funding acquisition, Formal analysis, Data curation, Conceptualization. **Samantha Jakimowicz:** Writing – review & editing, Supervision, Conceptualization. **Alison Robinson:** Writing – review & editing, Investigation. **Andrea McKittrick:** Writing – review & editing, Writing – original draft, Project administration, Formal analysis, Data curation, Conceptualization.

## Declaration of competing interest

This research was funded by a Charles Sturt University Early Researcher Grant.

## Figures and Tables

**Table 3:** JBI critical appraisal checklist for analytical cross-sectional studies.

**Cross sectional studies**																	
	(Alharbi, 2023)				(Anyango et al., 2024b)				(Alexander et al., 2025)				(Anyango et al., 2024a)				
	Yes	No	Unclear	n/a	Yes	No	Unclear	n/a	Yes	No	Unclear	n/a	Yes		No	Unclear	n/a
1. Were the criteria for inclusion in the sample clearly defined?	ⅹ				ⅹ				ⅹ				x				
2. Were the study subjects and the setting described in detail?	ⅹ				ⅹ				ⅹ				x				
3. Was the exposure measured in a valid and reliable way?			ⅹ		ⅹ				ⅹ				x				
4. Were objective, standard criteria used for measurement of the condition?	ⅹ				ⅹ				ⅹ				x				
5. Were confounding factors identified?			ⅹ		ⅹ					x			x				
6. Were strategies to deal with confounding factors stated?			ⅹ				ⅹ			x			x				
7. Were the outcomes measured in a valid and reliable way?	ⅹ					ⅹ			ⅹ				x				
8. Was appropriate statistical analysis used?	ⅹ					ⅹ			ⅹ				x				

**Cross Sectional Studies**																	
	(Cleary and Happell, 2005)				(Thongpriwan et al., 2015)				(Calma et al., 2022)				(Wareing et al., 2018)				
	Yes	No	Unclear	n/a	Yes	No	Unclear	n/a	Yes	No	Unclear	n/a	Yes		No	Unclear	n/a
1. Were the criteria for inclusion in the sample clearly defined?	ⅹ				ⅹ				ⅹ				ⅹ				
2. Were the study subjects and the setting described in detail?			ⅹ		ⅹ				ⅹ				ⅹ				
3. Was the exposure measured in a valid and reliable way?		ⅹ						ⅹ	ⅹ				ⅹ				
4. Were objective, standard criteria used for measurement of the condition?		ⅹ			ⅹ				ⅹ						ⅹ		
5. Were confounding factors identified?		ⅹ				ⅹ			ⅹ				ⅹ				
6. Were strategies to deal with confounding factors stated?		ⅹ						ⅹ	ⅹ						ⅹ		
7. Were the outcomes measured in a valid and reliable way?			ⅹ		ⅹ				ⅹ				ⅹ				
8. Was appropriate statistical analysis used?			ⅹ		ⅹ				ⅹ				ⅹ				

**Table 4:** JBI critical appraisal checklist for case control studies.

**Case Control Study**				
	(Bloomfield et al., 2015)			
	Yes	No	Unclear	n/a
1. Were the groups comparable other than the presence of disease in cases or the absence of disease in controls?	ⅹ			
2. Were cases and controls matched appropriately?	ⅹ			
3. Were the same criteria used for identification of cases and controls?	ⅹ			
4. Was exposure measured in a standard, valid and reliable way?	ⅹ			
5. Was exposure measured in the same way for cases and controls?	ⅹ			
6. Were confounding factors identified?	ⅹ			
7. Were strategies to deal with confounding factors stated?	ⅹ			
8. Were outcomes assessed in a standard, valid and reliable way for cases and controls?	ⅹ			
9. Was the exposure period of interest long enough to be meaningful?	ⅹ			
10. Was appropriate statistical analysis used?	ⅹ			

**Table 5:** JBI critical appraisal checklist for qualitative research.

**Qualitative Research**												
	(Abbey et al., 2006)				(Boyd-Turner et al., 2016)				(Foster et al., 2021)			
	Yes	No	Unclear	n/a	Yes	No	Unclear	n/a	Yes	No	Unclear	n/a
1. Is there congruity between the stated philosophical perspective and the research methodology?				ⅹ				ⅹ	ⅹ			
2. Is there congruity between the research methodology and the research question or objectives?	ⅹ				ⅹ				ⅹ			
3. Is there congruity between the research methodology and the methods used to collect data?	ⅹ				ⅹ				ⅹ			
4. Is there congruity between the research methodology and the representation and analysis of data?	ⅹ				ⅹ				ⅹ			
5. Is there congruity between the research methodology and the interpretation of results?	ⅹ				ⅹ				ⅹ			
6. Is there a statement locating the researcher culturally or theoretically?		ⅹ				ⅹ				ⅹ		
7. Is the influence of the researcher on the research, and vice- versa, addressed?	ⅹ				ⅹ				ⅹ			
8. Are participants, and their voices, adequately represented?	ⅹ				ⅹ				ⅹ			
9. Is the research ethical according to current criteria or, for recent studies, and is there evidence of ethical approval by an appropriate body?	ⅹ				ⅹ				ⅹ			
10. Do the conclusions drawn in the research report flow from the analysis, or interpretation, of the data?	ⅹ				ⅹ				ⅹ			

**Qualitative Research**												
	(Levett-Jones et al., 2007)				(McCall et al., 2009)				(McKenna et al., 2010)			
	Yes	No	Unclear	n/a	Yes	No	Unclear	n/a	Yes	No	Unclear	n/a
1. Is there congruity between the stated philosophical perspective and the research methodology?	ⅹ					ⅹ				ⅹ		
2. Is there congruity between the research methodology and the research question or objectives?	ⅹ				ⅹ				ⅹ			
3. Is there congruity between the research methodology and the methods used to collect data?	ⅹ				ⅹ				ⅹ			
4. Is there congruity between the research methodology and the representation and analysis of data?	ⅹ				ⅹ				ⅹ			
5. Is there congruity between the research methodology and the interpretation of results?	ⅹ				ⅹ				ⅹ			
6. Is there a statement locating the researcher culturally or theoretically?		ⅹ				ⅹ				ⅹ		
7. Is the influence of the researcher on the research, and vice- versa, addressed?			ⅹ			ⅹ			ⅹ			
8. Are participants, and their voices, adequately represented?	ⅹ				ⅹ				ⅹ			
9. Is the research ethical according to current criteria or, for recent studies, and is there evidence of ethical approval by an appropriate body?	ⅹ				ⅹ				ⅹ			
10. Do the conclusions drawn in the research report flow from the analysis, or interpretation, of the data?	ⅹ				ⅹ				ⅹ			

**Qualitative Research**												
	(Neill and Taylor, 2002)				(Peters et al., 2015)				(Anyango et al., 2024b)			
	Yes	No	Unclear	n/a	Yes	No	Unclear	n/a	Yes	No	Unclear	n/a
1. Is there congruity between the stated philosophical perspective and the research methodology?		ⅹ				ⅹ			ⅹ			
2. Is there congruity between the research methodology and the research question or objectives?			ⅹ		ⅹ				ⅹ			
3. Is there congruity between the research methodology and the methods used to collect data?			ⅹ		ⅹ				ⅹ			
4. Is there congruity between the research methodology and the representation and analysis of data?			ⅹ		ⅹ				ⅹ			
5. Is there congruity between the research methodology and the interpretation of results?		ⅹ			ⅹ				ⅹ			
6. Is there a statement locating the researcher culturally or theoretically?		ⅹ			ⅹ					ⅹ		
7. Is the influence of the researcher on the research, and vice- versa, addressed?		ⅹ			ⅹ					ⅹ		
8. Are participants, and their voices, adequately represented?		ⅹ			ⅹ						ⅹ	
9. Is the research ethical according to current criteria or, for recent studies, and is there evidence of ethical approval by an appropriate body?		ⅹ			ⅹ					ⅹ		
10. Do the conclusions drawn in the research report flow from the analysis, or interpretation, of the data?		ⅹ			ⅹ				ⅹ			

**Qualitative Research**								
	(Edmonds and Abdulmohdi, 2025)				(Lea et al., 2008)			
	Yes	No	Unclear	n/a	Yes	No	Unclear	n/a
1. Is there congruity between the stated philosophical perspective and the research methodology?	ⅹ				ⅹ			
2. Is there congruity between the research methodology and the research question or objectives?	ⅹ				ⅹ			
3. Is there congruity between the research methodology and the methods used to collect data?	ⅹ				ⅹ			
4. Is there congruity between the research methodology and the representation and analysis of data?	ⅹ				ⅹ			
5. Is there congruity between the research methodology and the interpretation of results?	ⅹ				ⅹ			
6. Is there a statement locating the researcher culturally or theoretically?		ⅹ			ⅹ			
7. Is the influence of the researcher on the research, and vice- versa, addressed?		ⅹ			ⅹ			
8. Are participants, and their voices, adequately represented?	ⅹ					ⅹ		
9. Is the research ethical according to current criteria or, for recent studies, and is there evidence of ethical approval by an appropriate body?			ⅹ			ⅹ		
10. Do the conclusions drawn in the research report flow from the analysis, or interpretation, of the data?	ⅹ				ⅹ			

**Table 6:** JBI Critical Appraisal Checklist for cohort studies.

**Cohort Studies**												
	(Patterson et al., 2018)				(Happell, 2008)				(Hunter et al., 2015)			
	Yes	No	Unclear	n/a	Yes	No	Unclear	n/a	Yes	No	Unclear	n/a
1. Were the two groups similar and recruited from the same population?	ⅹ							ⅹ				ⅹ
2. Were the exposures measured similarly to assign people to both exposed and unexposed groups?			ⅹ					ⅹ				ⅹ
3. Was the exposure measured in a valid and reliable way?	ⅹ								ⅹ			
4. Were confounding factors identified?	ⅹ					ⅹ			ⅹ			
5. Were strategies to deal with confounding factors stated?		ⅹ						ⅹ	ⅹ			ⅹ
6. Were the groups/participants free of the outcome at the start of the study (or at the moment of exposure)?	ⅹ						ⅹ			ⅹ		
7. Were the outcomes measured in a valid and reliable way?	ⅹ								ⅹ			
8. Was the follow up time reported and sufficient to be long enough for outcomes to occur?	ⅹ						ⅹ				ⅹ	
9. Was follow up complete, and if not, were the reasons to loss to follow up described and explored?	ⅹ								ⅹ			
10. Were strategies to address incomplete follow up utilized?				ⅹ			ⅹ				ⅹ	
11. Was appropriate statistical analysis used?	ⅹ										ⅹ	

**Cohort Studies**								
	(Kloster et al., 2007)				(Lim et al., 2020)			
	Yes	No	Unclear	n/a	Yes	No	Unclear	n/a
1. Were the two groups similar and recruited from the same population?	ⅹ					ⅹ		
2. Were the exposures measured similarly to assign people to both exposed and unexposed groups?	ⅹ					ⅹ		
3. Was the exposure measured in a valid and reliable way?	ⅹ					ⅹ		
4. Were confounding factors identified?		ⅹ				ⅹ		
5. Were strategies to deal with confounding factors stated?		ⅹ				ⅹ		
6. Were the groups/participants free of the outcome at the start of the study (or at the moment of exposure)?	ⅹ					ⅹ		
7. Were the outcomes measured in a valid and reliable way?	ⅹ					ⅹ		
8. Was the follow up time reported and sufficient to be long enough for outcomes to occur?	ⅹ					ⅹ		
9. Was follow up complete, and if not, were the reasons to loss to follow up described and explored?	ⅹ					ⅹ		
10. Were strategies to address incomplete follow up utilized?		ⅹ						ⅹ
11. Was appropriate statistical analysis used?	ⅹ					ⅹ		

**Table 7:** Mixed methods appraisal Tool (MMAT), version 2018.

**Category of study designs**	**Methodological quality criteria**	(Andrews et al., 2005)			(8)		
		Yes	No	Can’t tell	Yes	No	Can’t tell
1. Qualitative	1.1. Is the qualitative approach appropriate to answer the research question?				ⅹ		
	1.2. Are the qualitative data collection methods adequate to address the research question?				ⅹ		
	1.3. Are the findings adequately derived from the data?				ⅹ		
	1.4. Is the interpretation of results sufficiently substantiated by data?				ⅹ		
	1.5. Is there coherence between qualitative data sources, collection, analysis and interpretation?				ⅹ		
2. Quantitative randomized controlled trials	2.1. Is randomization appropriately performed?						
	2.2. Are the groups comparable at baseline?						
	2.3. Are there complete outcome data?						
	2.4. Are outcome assessors blinded to the intervention provided?						
	2.5 Did the participants adhere to the assigned intervention?						
3. Quantitative non-randomized	3.1. Are the participants representative of the target population?						
	3.2. Are measurements appropriate regarding both the outcome and intervention (or exposure)?						
	3.3. Are there complete outcome data?						
	3.4. Are the confounders accounted for in the design and analysis?						
	3.5. During the study period, is the intervention administered (or exposure occurred) as intended?						
4. Quantitative descriptive	4.1. Is the sampling strategy relevant to address the research question?						
	4.2. Is the sample representative of the target population?						
	4.3. Are the measurements appropriate?						
	4.4. Is the risk of nonresponse bias low?						
	4.5. Is the statistical analysis appropriate to answer the research question?						
5. Mixed methods	5.1. Is there an adequate rationale for using a mixed methods design to address the research question?	ⅹ					
	5.2. Are the different components of the study effectively integrated to answer the research question?	ⅹ					
	5.3. Are the outputs of the integration of qualitative and quantitative components adequately interpreted?	ⅹ					
	5.4. Are divergences and inconsistencies between quantitative and qualitative results adequately addressed?	ⅹ					
	5.5. Do the different components of the study adhere to the quality criteria of each tradition of the methods involved?	ⅹ					

**Category of study designs**	**Methodological quality criteria**	(Brown et al., 2008)					
		Yes	No	Can’t tell	Yes	No	Can’t tell
1. Qualitative	1.1. Is the qualitative approach appropriate to answer the research question?						
	1.2. Are the qualitative data collection methods adequate to address the research question?						
	1.3. Are the findings adequately derived from the data?						
	1.4. Is the interpretation of results sufficiently substantiated by data?						
	1.5. Is there coherence between qualitative data sources, collection, analysis and interpretation?						
2. Quantitative randomized controlled trials	2.1. Is randomization appropriately performed?						
	2.2. Are the groups comparable at baseline?						
	2.3. Are there complete outcome data?						
	2.4. Are outcome assessors blinded to the intervention provided?						
	2.5 Did the participants adhere to the assigned intervention?						
3. Quantitative non-randomized	3.1. Are the participants representative of the target population?						
	3.2. Are measurements appropriate regarding both the outcome and intervention (or exposure)?						
	3.3. Are there complete outcome data?						
	3.4. Are the confounders accounted for in the design and analysis?						
	3.5. During the study period, is the intervention administered (or exposure occurred) as intended?						
4. Quantitative descriptive	4.1. Is the sampling strategy relevant to address the research question?						
	4.2. Is the sample representative of the target population?						
	4.3. Are the measurements appropriate?						
	4.4. Is the risk of nonresponse bias low?						
	4.5. Is the statistical analysis appropriate to answer the research question?						
5. Mixed methods	5.1. Is there an adequate rationale for using a mixed methods design to address the research question?	ⅹ					
	5.2. Are the different components of the study effectively integrated to answer the research question?	ⅹ					
	5.3. Are the outputs of the integration of qualitative and quantitative components adequately interpreted?	ⅹ					
	5.4. Are divergences and inconsistencies between quantitative and qualitative results adequately addressed?			ⅹ			
	5.5. Do the different components of the study adhere to the quality criteria of each tradition of the methods involved?	ⅹ					

Category of study designs	Methodological quality criteria	(Gale et al., 2016)			(Lea et al., 2016)		
		Yes	No	Can’t tell	Yes	No	Can’t tell
1. Qualitative	1.1. Is the qualitative approach appropriate to answer the research question?						
	1.2. Are the qualitative data collection methods adequate to address the research question?						
	1.3. Are the findings adequately derived from the data?						
	1.4. Is the interpretation of results sufficiently substantiated by data?						
	1.5. Is there coherence between qualitative data sources, collection, analysis and interpretation?						
2. Quantitative randomized controlled trials	2.1. Is randomization appropriately performed?						
	2.2. Are the groups comparable at baseline?						
	2.3. Are there complete outcome data?						
	2.4. Are outcome assessors blinded to the intervention provided?						
	2.5 Did the participants adhere to the assigned intervention?						
3. Quantitative non-randomized	3.1. Are the participants representative of the target population?						
	3.2. Are measurements appropriate regarding both the outcome and intervention (or exposure)?						
	3.3. Are there complete outcome data?						
	3.4. Are the confounders accounted for in the design and analysis?						
	3.5. During the study period, is the intervention administered (or exposure occurred) as intended?						
4. Quantitative descriptive	4.1. Is the sampling strategy relevant to address the research question?						
	4.2. Is the sample representative of the target population?						
	4.3. Are the measurements appropriate?						
	4.4. Is the risk of nonresponse bias low?						
	4.5. Is the statistical analysis appropriate to answer the research question?						
5. Mixed methods	5.1. Is there an adequate rationale for using a mixed methods design to address the research question?		ⅹ		ⅹ		
	5.2. Are the different components of the study effectively integrated to answer the research question?	ⅹ			ⅹ		
	5.3. Are the outputs of the integration of qualitative and quantitative components adequately interpreted?		ⅹ		ⅹ		
	5.4. Are divergences and inconsistencies between quantitative and qualitative results adequately addressed?			ⅹ	ⅹ		
	5.5. Do the different components of the study adhere to the quality criteria of each tradition of the methods involved?		ⅹ		ⅹ		

**Category of study designs**	**Methodological quality criteria**	(O'Brien et al., 2008)			(Salamonson et al., 2018)		
		Yes	No	Can’t tell	Yes	No	Can’t tell
1. Qualitative	1.1. Is the qualitative approach appropriate to answer the research question?						
	1.2. Are the qualitative data collection methods adequate to address the research question?						
	1.3. Are the findings adequately derived from the data?						
	1.4. Is the interpretation of results sufficiently substantiated by data?						
	1.5. Is there coherence between qualitative data sources, collection, analysis and interpretation?						
2. Quantitative randomized controlled trials	2.1. Is randomization appropriately performed?						
	2.2. Are the groups comparable at baseline?						
	2.3. Are there complete outcome data?						
	2.4. Are outcome assessors blinded to the intervention provided?						
	2.5 Did the participants adhere to the assigned intervention?						
3. Quantitative non-randomized	3.1. Are the participants representative of the target population?						
	3.2. Are measurements appropriate regarding both the outcome and intervention (or exposure)?						
	3.3. Are there complete outcome data?						
	3.4. Are the confounders accounted for in the design and analysis?						
	3.5. During the study period, is the intervention administered (or exposure occurred) as intended?						
4. Quantitative descriptive	4.1. Is the sampling strategy relevant to address the research question?						
	4.2. Is the sample representative of the target population?						
	4.3. Are the measurements appropriate?						
	4.4. Is the risk of nonresponse bias low?						
	4.5. Is the statistical analysis appropriate to answer the research question?						
5. Mixed methods	5.1. Is there an adequate rationale for using a mixed methods design to address the research question?		ⅹ		ⅹ		
	5.2. Are the different components of the study effectively integrated to answer the research question?			ⅹ	ⅹ		
	5.3. Are the outputs of the integration of qualitative and quantitative components adequately interpreted?			ⅹ	ⅹ		
	5.4. Are divergences and inconsistencies between quantitative and qualitative results adequately addressed?			ⅹ			ⅹ
	5.5. Do the different components of the study adhere to the quality criteria of each tradition of the methods involved?			ⅹ			ⅹ

**Category of study designs**	**Methodological quality criteria**	(Wareing et al., 2017)			(Williamson et al., 2023)		
		Yes	No	Can’t tell	Yes	No	Can’t tell
1. Qualitative	1.1. Is the qualitative approach appropriate to answer the research question?						
	1.2. Are the qualitative data collection methods adequate to address the research question?						
	1.3. Are the findings adequately derived from the data?						
	1.4. Is the interpretation of results sufficiently substantiated by data?						
	1.5. Is there coherence between qualitative data sources, collection, analysis and interpretation?						
2. Quantitative randomized controlled trials	2.1. Is randomization appropriately performed?						
	2.2. Are the groups comparable at baseline?						
	2.3. Are there complete outcome data?						
	2.4. Are outcome assessors blinded to the intervention provided?						
	2.5 Did the participants adhere to the assigned intervention?						
3. Quantitative non-randomized	3.1. Are the participants representative of the target population?						
	3.2. Are measurements appropriate regarding both the outcome and intervention (or exposure)?						
	3.3. Are there complete outcome data?						
	3.4. Are the confounders accounted for in the design and analysis?						
	3.5. During the study period, is the intervention administered (or exposure occurred) as intended?						
4. Quantitative descriptive	4.1. Is the sampling strategy relevant to address the research question?						
	4.2. Is the sample representative of the target population?						
	4.3. Are the measurements appropriate?						
	4.4. Is the risk of nonresponse bias low?						
	4.5. Is the statistical analysis appropriate to answer the research question?						
5. Mixed methods	5.1. Is there an adequate rationale for using a mixed methods design to address the research question?	ⅹ			ⅹ		
	5.2. Are the different components of the study effectively integrated to answer the research question?	ⅹ			ⅹ		
	5.3. Are the outputs of the integration of qualitative and quantitative components adequately interpreted?	ⅹ			ⅹ		
	5.4. Are divergences and inconsistencies between quantitative and qualitative results adequately addressed?			ⅹ	ⅹ		
	5.5. Do the different components of the study adhere to the quality criteria of each tradition of the methods involved?	ⅹ			ⅹ		

**Table 8:** JBI checklist for quasi-experimental studies.

**Quasi-experimental study**											
		(Courtney et al., 2002)									
Internal Validity		Yes		No		Unclear				N/A	
1	Is it clear in the study what is the “cause” and what is the “effect” (i.e. there is no confusion about which variable comes first)?	☒		☐		☐				☐	
2	Was there a control group?	☐		☒		☐				☐	
3	Were participants included in any comparisons similar?	☐		☐		☐				☒	
4	Were the participants included in any comparisons receiving similar treatment/care, other than the exposure or intervention of interest?	☐		☐		☐				☒	
5	Were there multiple measurements of the outcome, both pre and post the intervention/exposure?	Yes		No		Unclear			N/A		
	Outcome 1 Impact on intention	☐		☒		☐			☐		
	Outcome 2 Further employment	☐		☒		☐			☐		
6	Were the outcomes of participants included in any comparisons measured in the same way?	Yes		No		Unclear			N/A		
	Outcome 1	☒		☐		☐			☐		
	Outcome 2	☒		☐		☐			☐		
7	Were outcomes measured in a reliable way?	Yes		No		Unclear			N/A		
	Outcome 1	☒		☐		☐			☐		
	Outcome 2	☒		☐		☐			☐		
**8**	Was follow-up complete and if not, were differences between groups in terms of their follow-up adequately described and analyzed?										
	Outcome 1	Yes		No		Unclear			N/A		
	Result 1	☐		☐		☐			☒		
	Result 2	☐		☐		☐			☒		

## References

[bib0001] Abbey J. (2006). Clinical placements in residential aged care facilities: the impact on nursing students' perception of aged care and the effect on career plans. Aust. J. Adv. Nurs..

[bib0002] Adams R. (2021). Understanding the factors that affect retention within the mental health nursing workforce: a systematic review and thematic synthesis. Int. J. Ment. Health Nurs..

[bib0003] Adeboye O.R. (2025). Perceptions of care homes as practice learning environments for pre-registration nursing students: a systematic-narrative hybrid literature review. Nurse Educ. Today.

[bib0004] AHPRA (2024). Fact sheet: what do nurses and midwives do?. https://www.nursingmidwiferyboard.gov.au/Codes-Guidelines-Statements/FAQ/Fact-sheet-What-do-nurses-and-midwives-do.

[bib0005] Alexander L. (2025). I felt like I was invisible’: nursing students' Experiences of mental health clinical placement—a cross-sectional study. Int. J. Ment. Health Nurs..

[bib0006] Alharbi T.A.F. (2023). Nursing students' perceptions on community care and placement preference in Saudi Arabian universities: a multicenter cross-sectional study. Nurse Educ. Today.

[bib0007] Andrews G. (2005). Place (ment) matters: students’ clinical experiences and their preferences for first employers. Int. Nurs. Rev..

[bib0008] Anyango E. (2024). An examination of the career decision-making self-efficacy of final-year nursing students. Nurse Educ. Today.

[bib0010] Anyango E. (2024). I changed my mind after my placement”: the influence of clinical placement environment on career choices of final-year pre-registration nurses. Collegian.

[bib0009] Anyango E. (2024). The impact of final-year clinical placements on nursing students' career planning for the graduate year and beyond. Nurse Educ Pr..

[bib0011] Anyango E. (2024). The influence of individual factors on the career preferences and specialty choices of final-year nursing students. J. Prof. Nurs..

[bib0012] Beks H. (2018). When you're it': a qualitative study exploring the rural nurse experience of managing acute mental health presentations. Rural. Remote Health.

[bib0013] Bloomfield J.G. (2015). Nursing students’ intentions to enter primary health care as a career option: findings from a national survey. Collegian.

[bib0014] Bourke S.L. (2021). Health care experiences in rural, remote, and metropolitan areas of Australia. Online J. Rural Nurs. Health Care.

[bib0015] Boyd-Turner D. (2016). The influence student placement experience can have on the employment choices of graduates: a paediatric nursing context. Nurse Educ Pr..

[bib0016] Braun V. (2024). Advanced Research Methods For Applied Psychology.

[bib0017] Brown J. (2008). Transforming students’ views of gerontological nursing: realising the potential of ‘enriched’environments of learning and care: a multi-method longitudinal study. Int. J. Nurs. Stud..

[bib0018] Calma K.R.B. (2022). Confidence, interest and intentions of final-year nursing students regarding employment in general practice. Collegian.

[bib0019] Campbell M. (2020). Synthesis without meta-analysis (SWiM) in systematic reviews: reporting guideline. BMJ.

[bib0020] Cleary M., Happell B. (2005). Recruitment and retention initiatives: nursing students’ satisfaction with clinical experience in the mental health field. Nurse Educ Pr..

[bib0021] Cottle-Quinn A. (2023). Factors that impact Australian early career nurses' intentions to remain in their position and the profession: a prospective cohort study. J. Nurs. Manage.

[bib0022] Courtney M. (2002). The impact of rural clinical placement on student nurses' employment intentions. Collegian.

[bib0023] Edmonds M., Abdulmohdi N. (2025). Final-year nursing students' placement experiences in a critical care setting: a qualitative study. Nurs. Crit. Care.

[bib0024] Fortabong A.A. (2025). Rural nursing workforce sustainability in Australia: a scoping review of global retention strategies. Aust. J. Rural Health.

[bib0025] Foster K. (2021). Anyone can have a mental illness’: a qualitative inquiry of pre-registration nursing students’ experiences of traditional mental health clinical placements. Int. J. Ment. Health Nurs..

[bib0026] Furber G. (2015). Preventing mental illness: closing the evidence-practice gap through workforce and services planning. BMC Health v Res..

[bib0027] Gale J. (2016). The experiences of student nurses on placements with practice nurses: a pilot study. Nurse Educ Pr..

[bib0028] Gatchel R.J. (2018). The importance of the profession of nursing in health care: current and future issues. J. Appl. Biobehav. Res..

[bib0029] Griffin K. (2026). The impact of mental health placement experiences pathway preferences for nursing students. Teach. Learn. Nurs..

[bib0030] Han R.M. (2018). Relationships among factors affecting advanced practice registered nurses' job satisfaction and intent to leave: a systematic review. J. Am. Assoc. Nurs. Pr..

[bib0031] Happell, B. (2008). Changing their minds: using clinical experience to'convert'undergraduate nursing students to psychiatric nursing.

[bib0032] Hong Q. (2018). Mixed methods appraisal tool (MMAT) version 2018. http://mixedmethodsappraisaltoolpublic.pbworks.com/w/file/fetch/127916259/MMAT_2018_criteria-manual_2018-08-01_ENG.pdf.

[bib0033] Hunter L. (2015). Nursing students’ attitudes about psychiatric mental health nursing. Issues Ment. Health Nurs..

[bib0034] Hurley J. (2009). A qualitative study of mental health nurse identities: many roles, one profession. Int. J. Ment. Health Nurs..

[bib0035] Jirwe M., Rudman A. (2012). Why choose a career in nursing?. J. Adv. Nurs..

[bib0036] Kloster T. (2007). Nursing students’ career preferences: a Norwegian study. J. Adv. Nurs..

[bib0037] Lea E. (2016). Aspects of nursing student placements associated with perceived likelihood of working in residential aged care. J. Clin. Nurs..

[bib0038] Lea J. (2008). The lure of the bush: do rural placements influence student nurses to seek employment in rural settings?. Collegian.

[bib0039] Levett-Jones T. (2007). Belongingness: a montage of nursing students’ stories of their clinical placement experiences. Contemp. Nurse.

[bib0040] Lim H.J. (2020). Students' mental health clinical placements, clinical confidence and stigma surrounding mental illness: a correlational study. Nurs. Educ. Today.

[bib0041] Liu X.-L. (2023). Factors influencing retention among regional, rural and remote undergraduate nursing students in Australia: a systematic review of current research evidence. Int. J. Env. Res. Public Health.

[bib0042] McCall L. (2009). Influence of clinical placement on undergraduate midwifery students’ career intentions. Midwifery.

[bib0043] McKenna L. (2010). Clinical placements and nursing students' career planning: a qualitative exploration. Int. J. Nurs. Pr..

[bib0044] Neill J., Taylor K. (2002). Undergraduate nursing students ‘clinical experiences in rural and remote areas: recruitment implications. Aust. J. Rural Health.

[bib0045] O'Brien L. (2008). Improving the undergraduate clinical placement experience in mental health nursing. Issues Ment. Health Nurs..

[bib0046] Page M.J. (2021). The PRISMA 2020 statement: an updated guideline for reporting systematic reviews. BMJ.

[bib0047] Patterson C. (2018). Mental health nursing placement: a comparative study of non-traditional and traditional placement. Nurs. Educ Pr..

[bib0048] Peters K. (2015). Nursing students’ experiences of clinical placement in community settings: a qualitative study. Collegian.

[bib0049] Peters M. (2023). Time to solve persistent, pernicious and widespread nursing workforce shortages. Int. Nurs. Rev..

[bib0050] Salamonson Y. (2018). Does clinical placement in a community alcohol and other drugs rehabilitation centre influence students’ career intention?. Int. J. Ment. Health Nurs..

[bib0051] Tamata A.T., Mohammadnezhad M. (2023). A systematic review study on the factors affecting shortage of nursing workforce in the hospitals. Nurs. Open.

[bib0052] Thongpriwan V. (2015). Undergraduate nursing students’ attitudes toward mental health nursing. Nurse Educ. Today.

[bib0053] Tran M. (2024). Health workforce shortage in australia post covid-19-international perspectives. Aust. Natl. Univ. Natl. Cent. Health Workforce Stud.: Health Workforce Data Insights-Mar 2024.

[bib0054] Wareing M. (2017). The influence of placements on adult nursing graduates' choice of first post. Br. J. Nurs..

[bib0055] Wareing M. (2018). Impact of clinical placements on graduates' choice of first staff-nurse post. Br. J. Nurs..

[bib0056] Wiggins D. (2022). Factors that influence scope of practice of the five largest health care professions in Australia: a scoping review. Hum. Resour. Health.

[bib0057] Williamson G.R. (2023). Student nurses as a future general practice nursing workforce. Implementing collaborative learning in practice: implications for placement learning and patient access. A mixed methods study. BMC Nurs..

[bib0058] Wilson A. (2026). Pre-registration nursing student experiences of international mental health clinical placement: a scoping review with relevance to the Australian context. Int. J. Ment. Health Nurs..

[bib0059] Wu L. (2015). Why not nursing? A systematic review of factors influencing career choice among healthcare students. Int. Nurs. Rev..

[bib0060] Yang S. (2024). Paper presented at the 4th International Conference on Public Administration, Health and Humanity Development (PAHHD 2024).

